# Reproductive outcomes of dual trigger with combination GnRH agonist and hCG versus trigger with hCG alone in women undergoing IVF/ICSI cycles: a retrospective cohort study with propensity score matching

**DOI:** 10.1186/s12884-022-04899-2

**Published:** 2022-07-22

**Authors:** Li Dong, Fang Lian, Haicui Wu, Shan Xiang, Yuan Li, Chaofeng Wei, Xiaona Yu, Xin Xin

**Affiliations:** 1 Shandong University of Traditional Chinese Medicine, Jinan, China; 2 Affiliated Hospital of Shandong University of Traditional Chinese Medicine, Jinan, China

**Keywords:** Dual trigger, GnRH antagonist protocols, Fresh embryo transfer, Laboratory outcomes, Clinical outcomes, Propensity score matching

## Abstract

**Background:**

Despite a large number of studies on the selection of trigger drugs, it remains unclear whether the dual trigger with human chorionic gonadotropin (hCG) and gonadotropin-releasing hormone (GnRH) agonist, compared to the trigger with hCG alone, can improve the reproductive outcome of patients undergoing assisted reproductive technology. Therefore, this study aimed to compare the laboratory and clinical outcomes of dual trigger versus single trigger.

**Methods:**

In this retrospective cohort study, we evaluated 520 in vitro fertilization/intracytoplasmic sperm injection (IVF/ICSI) cycles between July 2014 and September 2020 at the Reproductive and Genetic Center of Integrative Medicine, The Affiliated Hospital of Shandong University of Traditional Chinese Medicine. All patients underwent IVF/ICSI treatment with fresh embryo transfer using the GnRH antagonist protocol. We used propensity score matching to control for confounding variables and binary logistic regression analysis to determine the correlations between trigger methods and pregnancy outcomes. After propensity score matching, 57 cycles from each group were evaluated and compared for laboratory or clinical outcomes in this retrospective cohort study.

**Results:**

There was no significant difference in the number of oocytes retrieved, embryos available, top-quality embryos, or the rate of normal fertilization between the dual-trigger and single-trigger protocols, respectively. The incidence of ovarian hyperstimulation syndrome, implantation rate, biochemical pregnancy rate, clinical pregnancy rate, ectopic pregnancy rate, early miscarriage rate, and live birth rate were also similar between the two groups, while the miscarriage rate (37.0% vs. 12.5%, *p* = 0.045) was higher in the dual-trigger than the single-trigger group. Subsequent binary logistic regression analysis showed that age was a remarkably significant independent predictor of both clinical pregnancy rate (odds ratio = 0.90, 95% confidence interval: 0.84–0.97, *p* = 0.006) and live birth rate (odds ratio = 0.89, 95% confidence interval: 0.82–0.97, *p* = 0.005).

**Conclusions:**

Therefore, dual-trigger for final oocyte maturation might increase miscarriage rate, but in terms of the laboratory and other pregnancy outcomes such as clinical pregnancy rate, early miscarriage rate or live birth rate, there was no evidence to show that dual trigger was superior to an hCG-trigger alone for patients undergoing GnRH-antagonist cycles with fresh embryo transfer.

**Trial registration:**

Retrospectively registered.

## Background

It has been more than four decades since the first test-tube baby, Louise Brown, was born via the application of assisted reproductive technology [1]. Since then, thousands of babies are born via in vitro fertilization (IVF) every year to women with infertility. Intracytoplasmic sperm injection (ICSI) technology emerged in the early 1990s [2] and has fulfilled the desires of male patients suffering from severely compromised fertility to father children. Owing to its safety and efficiency after extensive research and development, the gonadotrophin-releasing hormone (GnRH) antagonist protocol has been used as the mainstream approach for controlled ovarian hyperstimulation (COH), especially for high or poor ovarian responders [3]. Compared with the traditional long GnRH agonist protocol, GnRH antagonist protocol can effectively reduce the duration and dosage of gonadotrophin (Gn) treatment [4] and decrease the risk of ovarian hyperstimulation syndrome (OHSS) [4–6].

In the cycles that employ GnRH antagonist protocol, in addition to human chorionic gonadotropin (hCG) for induction of final maturation of the oocyte, GnRH agonists are also used; these were first introduced by Gonen 30 years ago [7]. Recent studies revealed that a single injection of GnRH agonist may be effective in preventing OHSS on account of the GnRH agonist that induces an endogenous release of luteinizing hormone (LH), which is more physiologic than hCG [8–10]. Previous randomized controlled trials (RCTs) reported poor clinical outcomes for the early pregnancy loss rate when a GnRH agonist was used to trigger ovulation [11]. However, these poor results could be improved by strengthening the luteal phase support (LPS) with progesterone and estradiol [12–14]. Therefore, it has been found that for high ovarian responders (HORs) undergoing fresh autologous blastocyst transfers, the dual trigger method combining GnRH agonist with low-dose hCG resulted in significantly improved clinical pregnancy outcomes compared to GnRH agonist trigger alone, despite an increased incidence of OHSS [15].

Recent relevant studies have discussed whether the dual trigger for final oocyte maturation in the GnRH antagonist protocol with fresh embryo transfer (ET) cycles can be more beneficial in improving laboratory indicators and pregnancy outcomes than triggered by using GnRH agonist or hCG alone. A Meta-analysis involving 1048 individuals showed that dual trigger significantly improved live birth rate (LBR) [16], meanwhile another retrospective cohort study in which 10,427 patients were included discovered that dual trigger also reduced the risk of OHSS in the context of facilitated oocyte maturation [17]. However, a single-blind RCT [18] and retrospective study [19] involving 192 and 469 normal ovarian responders, respectively, have shown that the dual-trigger is not an effective alternative for enhancing pregnancy outcomes, although it increased the number of oocytes and embryos obtained. Overall, there is still no consensus on whether dual trigger can effectively improve reproductive outcomes.

Given the preliminary studies, we thought that further investigation regarding the efficacy of dual trigger was warranted. Moreover, no well-matched cohort study has been conducted. Here we investigated whether a dual trigger for final oocyte maturation with a combination of a single dose of GnRH agonist and a standard dose of hCG could improve the reproductive outcomes compared with conventional hCG trigger alone in GnRH antagonist IVF/ICSI-fresh ET cycles.

## Methods

### Study design

In the present retrospective cohort study, a review of medical records from July 1, 2014, through September 30, 2020, was performed for all IVF/ICSI-fresh ET cycles that underwent the GnRH antagonist protocol at the Reproductive and Genetic Center of Integrative Medicine, The Affiliated Hospital of Shandong University of Traditional Chinese Medicine. The study received approval and informed consent waiver from the Ethics Committee of the Reproductive Medicine Ethics Committee of the Affiliated Hospital of the Shandong University of Traditional Chinese Medicine, which waived informed consent owing to the retrospective cohort study design.

### Inclusion and exclusion criteria

The inclusion criteria were women: (i) aged< 50 years; (ii) with a body mass index (BMI) of 18–35 kg/m^2^; and (iii) who received GnRH antagonist and underwent fresh ET during the IVF/ICSI cycles. Our exclusion criteria were women: (i) with other underlying diseases that could not tolerate childbearing; (ii) suffering from an endocrine disorder (diabetes mellitus, hyperprolactinemia, thyroid dysfunction, congenital adrenal hyperplasia, Cushing syndrome); (iii) with a history of recurrent spontaneous abortion; and (iv) with uterine anomaly, endometriosis, or chromosomal abnormalities that cause infertility. Most of the data were obtained from the patients’ IVF/ICSI cycle electronic medical records, and the missing data were processed by telephone follow-up and by querying the patients’ inpatient medical records at the time of oocyte retrieval.

After applying the inclusion and exclusion criteria, a total of 520 IVF/ICSI with fresh ET cycles were included and then divided into two groups: dual-trigger and hCG trigger. Applying either hCG alone or the dual-trigger for final oocyte maturation depended on the attending physician. According to the standard operating procedure established by the department, patients in the dual-trigger group either with a history of < 60% MII oocytes obtained in IVF/ICSI cycles or with uneven follicle development on the trigger day, and the ultimate goal of using dual trigger was to improve the oocyte maturation rate.

### Treatment protocols

All patients were treated with the GnRH antagonist protocol in the flexible mode for COH. On the second day of the menstrual cycle, baseline hormone levels, such as follicle-stimulating hormone (FSH), LH and estrogen (E_2_), were measured and transvaginal ultrasound for antral follicles was conducted to evaluate the baseline ovary status. If the conditions permitted, 150–225 IU/d of recombinant human follicle-stimulating hormone (rFSH, GONAL-F, Merck Serono, Darmstadt, Germany) with or without application of human menopausal gonadotropin (HMG, Lizhu Pharma, Shaoguan, China) was injected from the third day of the menstrual cycle, according to the age, BMI, antral follicle count, and previous response to COH. Besides, the addition of human recombinant luteinizing hormone (rLH, Luveris, Merck Serono) and recombinant human growth hormone (rhGH, Genheal, United Cell Biotechnology, Shanghai, China) during COH was at the physician’s discretion. The dosage of Gn was adjusted lying on the follicular growth and serum level of E_2_. When the dominant follicle reaches 12–14 mm in diameter, the GnRH antagonist 0.25 mg/d (Cetrotide, Merck Serono) was administered until the day of final oocyte maturation. The trigger was administered when at least two follicles reached a diameter of 18 mm or three follicles reached a diameter of 17 mm. The hCG trigger group (*n* = 458 patients) received 250 μg of recombinant hCG (Ovidrel, Merck Serono) alone, and the dual-trigger group (*n* = 62 patients) received 0.2 mg triptorelin (Decapeptyl, Ferring Pharmaceuticals, Saint-Prex, Switzerland) plus 250 μg of recombinant hCG. The oocyte was retrieved by transvaginal ultrasonography after 36–38 h. Whether fertilization was conducted by IVF or ICSI depended on the results of semen analysis or prior fertilization condition.

Normal fertilization was confirmed when two pronuclei and two polar bodies were observed after 16–18 h of insemination. Amphicytula were cultured in a cleavage medium (Cook Medical, Bloomington, IN, USA), and the embryo development was assessed daily (i.e., number, shape, evenness, and fragment rate of embryos). Fresh ET was performed 3 days after oocyte retrieval. The number of transferred embryos was 1–2 depending on the embryo quality and patient age. A high-quality embryo was defined as one that met the following three criteria established by the Istanbul consensus workshop [20]: (i) ≥6 cells 3 days after fertilization, (ii) < 10% fragmentation, and (iii) symmetric blastomeres. Then, the remaining embryos that did not satisfy the criteria were transferred and cultured to the blastocyst stage in the blastocyst medium (Cook Medical). Embryos that were not transferred were cryopreserved by vitrification.

All patients received 40 mg/d of progestin injection (Xianju, Taizhou, China) for LPS from the day of oocyte retrieval. The serum β-hCG level was measured 14 days after cleavage embryo transfer, and the pregnancy test was considered positive if the β-hCG level was ≥10 mIU/mL [21]. In case of a positive pregnancy test, the LPS strategy was continued until 10 weeks of gestation.

### Outcome measures

Laboratory and clinical data were collected as follows: the number of oocytes retrieved, rates of normal fertilization, number of embryos transferable, number of high-quality embryos, OHSS incidence, implantation rate (IR), biochemical pregnancy rate (BPR), clinical pregnancy rate (CPR), ectopic pregnancy rate (EPR), early miscarriage rate (EMR), miscarriage rate (MR), and LBR. Normal fertilization rate was defined as the number of normal fertilized oocytes divided by the total number of retrieved oocytes. The implantation rate was calculated as the number of gestational sacs divided by the total number of transferred embryos. Biochemical pregnancy was defined as a positive pregnancy test with no intrauterine or extrauterine gestational sac detected on vaginal ultrasound. Clinical pregnancy was defined as the presence of gestational sacs with fetal heartbeat on ultrasonogram 14 days after a positive pregnancy test. A diagnosis of ectopic pregnancy was made either by laparoscopy or sonographic visualization of an extrauterine gestational sac. Early miscarriage was defined as pregnancy loss that occurs spontaneously before 12 weeks of gestation [22]. Miscarriage refers to the termination of pregnancy before 28 weeks of gestation and a fetus weighing less than 1000 g. The LBR was calculated by dividing the total deliveries of viable infants over 28 gestational weeks by the total number of fresh ET cycles.

### Statistical analysis

**Fig. 1:**
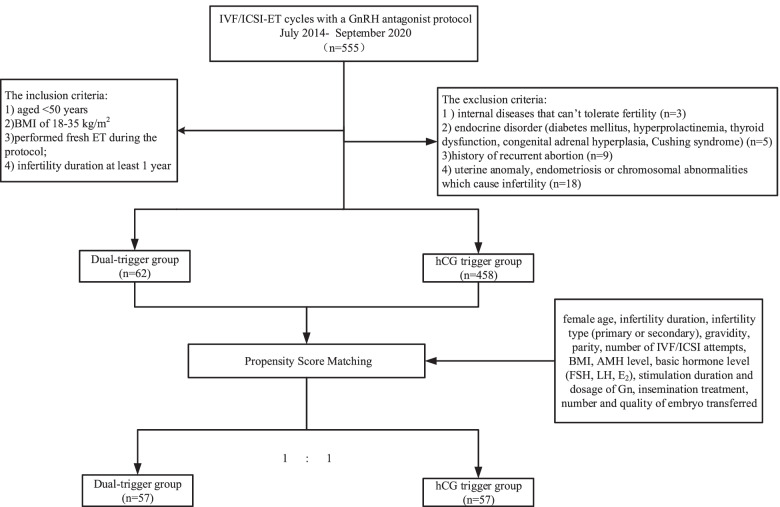
Study flow chart

Data analyses were carried out using SPSS version 22.0 (IBM Corp., Armonk, NY, USA). We compared laboratory and clinical outcomes for the two groups in a propensity score matching (PSM) cohort to minimize potential deviation (Fig. 1). The propensity scores were calculated using binary logistic regression analyses based on the following patient variables at baseline: patient age; infertility duration; infertility type (primary or secondary); gravidity; parity; number of previous IVF/ICSI attempts; BMI; level of anti-Mullerian hormone (AMH); basic hormone levels of FSH, LH, and E_2_; stimulation duration and dosage of Gn; insemination treatment; and the number and quality of embryos transferred. A 1:1 pair matching was performed using the caliper-matching method, and a 0.02 propensity score tolerance was imposed on the maximum propensity score distance. Shapiro–Wilk test was used to assess the normality of data distribution. Continuous variables were expressed as mean ± standard deviations (SD) and then compared using Student’s *t*-tests or Mann–Whitney U-tests. Categorical variables were presented as frequencies and percentages, and the between-group differences were analyzed using the chi-square test. *P* < 0.05 was considered statistically significant. We also used binary logistic regression analysis to assess the association between trigger protocols or other potential factors and pregnancy outcomes. We calculated the adjusted odds ratio (OR) with a 95% confidence interval (CI).

## Results

A total of 520 IVF/ICSI cycles using the GnRH antagonist protocol with fresh ET were analyzed in our study, including 62 cycles in the dual-trigger group and 458 cycles in the hCG trigger group. Simultaneously, 57 cycles were matched in a 1:1 ratio after PSM (Fig. 1). There were no significant differences between the two groups in patient characteristics after PSM (Table 1). The characteristics of ovarian stimulation and laboratory indicators for each group are presented in Table 2. Differences regarding the total dosage and duration of Gn stimulation; methods of fertilization; serum E_2_, LH, and P levels; and number of pre-ovulatory follicles > 14 mm at the day of triggering between the two groups were not found. Moreover, normal fertilization rate, number of oocytes retrieved, serum E_2_, P levels at the day of transfer, number of embryos available, number of top-quality embryos or the number and quality of embryos used for fresh ET were similar between the two groups.

**Table 1:** Comparison of patients’ baseline characteristics between groups

	Dual trigger (*n* = 57)	hCG trigger (*n* = 57)	*p*-value
Patient age (years)	35.09 ± 6.16	35.60 ± 5.89	0.653
Infertility duration (years)	3.33 ± 3.01	3.58 ± 3.76	0.701
Infertility type (%)			0.677
Primary	17/57 (29.8)	15/57 (26.3)	
Secondary	40/57 (70.2)	42/57 (73.7)	
Gravidity (n)	1.47 ± 1.48	1.68 ± 1.45	0.445
Parity (n)	0.47 ± 0.63	0.56 ± 0.71	0.486
BMI (kg/m^2^)	24.37 ± 3.86	24.58 ± 3.54	0.762
AMH (ng/mL)	2.29 ± 1.67	2.24 ± 1.62	0.886
Basal FSH (mIU/mL)	8.55 ± 2.87	8.97 ± 4.81	0.572
Basal LH (mIU/mL)	4.48 ± 2.48	4.68 ± 2.87	0.691
Basal E_2_ (pg/mL)	49.07 ± 42.19	66.97 ± 77.75	0.130
Previous IVF/ICSI attempts (n)	0.79 ± 1.42	0.44 ± 0.76	0.104

Data are presented as mean ± SD and proportion (%)

*AMH* Anti-Müllerian hormone, *BMI* Body mass index, *E*_*2*_ Estrogen, *FSH* Follicle-stimulating hormone, *ICSI* Intracytoplasmic sperm injection, *IVF* In vitro fertilization, *LH* Luteinizing hormone

**Table 2:** Characteristics of ovarian stimulation and IVF/ICSI cycle outcomes of each group

	Dual trigger (*n* = 57)	hCG trigger(n = 57)	*p*-value
Duration of stimulation (d)	9.95 ± 2.20	9.56 ± 2.49	0.382
Dosage of Gn (IU)	2552.85 ± 1017.63	2375.66 ± 963.93	0.342
Fertilization (%)			0.528
IVF	43/57 (75.4)	40/57 (70.2)	
ICSI	14/57 (24.6)	17/57 (29.8)	
No. of follicles > 14 mm on trigger day (n)	6.86 ± 3.55	6.40 ± 3.25	0.476
E_2_ on trigger day (pg/mL)	2027.37 ± 1168.73	2082.05 ± 1186.33	0.805
LH on trigger day (mIU/mL)	2.89 ± 2.23	3.33 ± 2.20	0.295
P on trigger day (ng/mL)	0.76 ± 0.42	0.78 ± 0.36	0.848
No. of oocytes retrieved (n)	7.04 ± 3.87	6.53 ± 3.36	0.455
Normal fertilization rate (%)	67.72 ± 25.63	65.54 ± 26.58	0.657
E_2_ on transfer day (pg/mL)	995.30 ± 644.26	964.70 ± 625.91	0.798
P on transfer day (ng/mL)	34.48 ± 8.64	36.38 ± 6.66	0.848
No. of embryos available (n)	2.81 ± 1.85	2.84 ± 1.73	0.191
No. of top-quality embryos (n)	0.84 ± 0.98	0.79 ± 1.05	0.782
No. of embryo transfer (n)	1.75 ± 0.43	1.77 ± 0.42	0.828
At least one top-quality embryo transfer (%)	26/57 (45.6)	31/57 (54.4)	0.349

Data are presented as mean ± SD and proportion (%)

*Gn* Gonadotrophin, *E*_*2*_ Estrogen, *ICSI* Intracytoplasmic sperm injection, *IVF* In vitro fertilization, *LH* Luteinizing hormone, *P* Progesterone

Pregnancy outcomes between the two groups are presented in Table 3. We found that the MR was increased in the dual-trigger group (37.0% vs. 12.5%, *p* = 0.045) compared to that in the hCG-trigger group. However, no significant differences were found between the dual-trigger and hCG-trigger groups in terms of IR, BPR, CPR, EPR, EMR, and LBR. There was one case of moderate OHSS in the hCG-trigger group and one case of mild OHSS in the dual-trigger group, neither of which required hospitalization. A binary logistic regression model was used to determine whether dual-trigger use had a greater effect on the CPR, EMR, MR, or LBR than the hCG-trigger group (Table 4). Otherwise, possible risk factors that may impact pregnancy outcomes such as age, BMI, basal FSH level, and the number of pre-ovulatory follicles > 14 mm on trigger day were included in the analysis. The results showed that age was a negative independent factor affecting CPR (OR = 0.90, 95% CI: 0.84–0.97, *p* = 0.006) and LBR (OR = 0.89, 95% CI: 0.82–0.97, *p* = 0.005). In addition, there were no statistically significant differences between the dual-trigger group and hCG-trigger group in terms of EMR, MR, CPR and LBR.

**Table 3:** Pregnancy outcomes for both groups

	Dual trigger (*n* = 57)	hCG trigger (*n* = 57)	*p*-value
OHSS rate (%)	1/57 (1.8)	1/57 (1.8)	1.000
Implantation rate (%)	30/100 (30.0)	26/101 (25.7)	0.501
Biochemical pregnancy rate (%)	15/57 (26.3)	11/57 (19.3)	0.372
Clinical pregnancy rate (%)	27/57 (47.4)	24/57 (42.1)	0.572
Ectopic pregnancy rate (%)	1/27 (3.7)	2/24 (8.3)	0.916
Early miscarriage rate (%)	8/27 (29.6)	2/24 (8.3)	0.081
Miscarriage rate (%)	10/27 (37.0)	3/24 (12.5)	0.045
Live birth rate (%)	15/57 (28.1)	20/57 (33.3)	0.542

Data are presented as proportion (%)

*OHSS* Ovarian hyperstimulation syndrome

**Table 4:** Analyses of factors affecting clinical pregnancy rate, early miscarriage rate, miscarriage rate, and live birth rate using binary logistic regression model

Variable	Clinical pregnancy rate (%)		Early miscarriage rate (%)		Miscarriage rate (%)		Live birth rate (%)	
	Adjusted OR (95% CI)	*p*-value	Adjusted OR (95% CI)	*p*-value	Adjusted OR (95% CI)	*p*-value	Adjusted OR (95% CI)	*p*-value
Dual vs. hCG trigger	1.18 (0.54–2.58)	0.678	4.62 (0.78–27.24)	0.091	4.05 (0.93–17.62)	0.062	0.73 (0.31–1.69)	0.462
Age (years)	0.90 (0.84–0.97)	0.006	1.15 (0.98–1.34)	0.093	1.04 (0.92–1.19)	0.517	0.89 (0.82–0.97)	0.005
BMI (kg/m^2^)	0.94 (0.84–1.04)	0.234	1.19 (0.97–1.47)	0.098	1.04 (0.88–1.24)	0.646	0.93 (0.82–1.04)	0.202
Basal FSH (IU/L)	1.02 (0.92–1.14)	0.669	1.01 (0.81–1.26)	0.916	0.99 (0.83–1.18)	0.911	1.03 (0.92–1.15)	0.582
No. of follicles > 14 mm on trigger day (n)	1.03 (0.91–1.16)	0.673	1.14 (0.89–1.46)	0.296	1.02 (0.82–1.26)	0.864	0.97 (0.85–1.11)	0.657

*BMI* Body mass index, *CI* Confidence interval, *FSH* Follicle-stimulating hormone, *OR* Odds ratio

## Discussion

Since the question of whether dual-trigger improves oocyte maturation and pregnancy outcomes has been raised in the past few years, numerous studies have been conducted, but as of today there are still no conclusive results. In this retrospective cohort study, we evaluated the IVF/ICSI laboratory and clinical results of dual-trigger versus hCG-trigger alone in GnRH antagonist cycles. Our results showed that dual-trigger using GnRH agonist and hCG were slightly superior to hCG-trigger alone in terms of the numbers of oocytes retrieved and high-quality embryos, but these differences were not significant. And there was no difference in the incidence of OHSS between the two groups. Additionally, the normal fertilization rate, IR, BPR, and CPR in the dual-trigger group were mildly higher and the LBR lower in the dual-trigger group than the hCG trigger-alone group; however, none of these differences were statistically significant. Our study demonstrated that the MR was higher in the dual-trigger group than in the hCG-alone group; this result could likely explain the above phenomenon that the higher IR and CPR, however a lower LBR in dual-trigger group than the hCG trigger-alone group. However, in terms of EMR, despite being higher in the dual-trigger group, it did not show a statistically significant difference. Binary logistic regression showed that age was identified as an independent risk factor for the CPR and LBR but not the EMR and MR. Undeniably, most studies showed that age is an independent risk factor for MR [23]. This study, however, found that age was not a risk factor for EMR, MR because of the small sample size, which increases the probability of type II errors, and may also be influenced by the mode of transplantation [24]. Although the previous statistical results indicated that dual-trigger caused high MR, and subsequent regression analysis suggested that dual-trigger could be a potential predictor of MR, it was not statistically significant (OR = 4.05, 95% CI: 0.93–17.62, *p* = 0.062). The above results suggested that the dual-trigger pattern causes a high rate of miscarriage, which still needs to be verified in a later clinical trial with a large sample.

Previous studies showed that in the GnRH antagonist IVF/ICSI-fresh ET treatment cycles, only using the GnRH agonist for final oocyte maturation can effectively prevent OHSS but also reduce the LBR to some extent [8, 25, 26] compared with hCG trigger alone. The GnRH agonist trigger would result in massive luteolysis [27], which likely occurs as a result of LH depletion from the pituitary and transient GnRH receptor down-regulation; all of these factors can cause the withdrawal of LH support for the corpus luteum [28]. Hence, relevant experts pointed out that “dual-trigger” strategy using a GnRH agonist and low-dose hCG can achieve the same effect on ongoing pregnancy rate as triggering by GnRH agonist alone with enhanced LPS, and superior to GnRH agonist alone with standard LPS [14].

Our results are consistent with previous studies that reported similar pregnancy outcomes from dual-trigger versus hCG trigger alone under GnRH antagonist scheme [18, 19, 29]. In Şükür et al.’s retrospective study, the number of high-quality embryos in dual-trigger cycles was relatively high and was similar to the number of oocytes retrieved or MII oocytes retrieved. However, Gao et al.’s retrospective study and Eftekhar et al.’s RCT reported that the number of oocytes retrieved and embryos available were both higher in the dual-trigger group than the hCG trigger-alone group. A 2021 systematic review and meta-analysis similarly found that the two trigger methods were equally effective in terms of pregnancy rate [30]. At variance with the findings of our study, another meta-analysis reported that although the number of mature oocytes or fertilized oocytes retrieved and the ongoing pregnancy rate were similar, the CPR with dual-trigger was significantly higher than hCG trigger alone [31]. In an even larger systematic review and meta-analysis with 1048 participants, the authors reported the dual-trigger group had a significantly higher LBR than the hCG trigger-alone group [16]. In addition, a retrospective study by Lin and collaborators (*n* = 427 patients) [32] confirmed that dual trigger significantly improved the CPR, LBR and reduced the MR in women with diminished ovarian reserve during GnRH antagonist cycles. Another RCT (*n* = 221 patients) [33] reported that dual trigger markedly increased overall and ongoing pregnancy rates in completed cycles but not in all initiated cycles, and investigator Schachter speculated that the results probably were correlated with GnRH antagonist schemes affecting endometrial GnRH receptors. Unlike our study, the above-mentioned researches focused mainly on the CPR or LBR, whereas we found that dual-trigger could cause a higher MR than hCG-trigger alone. The discrepancies between our study and the other studies are due (at least in part) to the variation in ovarian response of included patients, the small sample size, or the differences in selection for COH, trigger methods and LPS. Encouraged by our preliminary results, we plan to increase the sample size in future investigations and analyze clinical outcomes after classifying patients according to ovarian response to obtain more accurate evidence-based data.

In our study, the number of oocytes retrieved, embryos available, and high-quality embryos were similar in both groups. In accordance with our results, some studies reached similar conclusions concerning dual-trigger versus hCG-trigger [29, 34]. An RCT including 155 patients found that there were remarkable enhancements on the number of oocytes retrieved, MII oocytes, total number of blastocysts, and high-quality blastocysts transferred; moreover, the CPR and LBR per transfer were also significantly higher in the dual-trigger group compared to the hCG trigger-alone group in normal responders [35]. Interestingly, some retrospective cohort studies evaluated the impact of dual-trigger strategy for oocyte maturation in patients with a previous history of a high rate of immature oocyte retrieval [36–38]. The results of these studies clearly demonstrate that the dual-trigger protocol was efficient to improve the rate of retrieval of mature oocytes. However, some studies had a contradictory view on pregnancy outcomes. Griffin and associates believed that the reproductive outcomes will still be poor because of an underlying oocyte dysfunction [37], and other investigators held the view that dual-trigger can simultaneously enhance the CPR [36, 38]. Thus, the benefits of a dual-trigger for oocyte maturation and even the pregnancy outcomes in patients are still controversial, which also underscores the need for large-scale, multicenter RCTs to validate these findings.

In recent years, researchers have reported the discrepancy of cycle results between two trigger modes in different ovarian reactions such as HOR and poor ovarian responder (POR). Multiple retrospective cohort studies [39–41] or systematic review and meta-analyses [42] present different views regarding the outcomes of dual-trigger on PORs. After considering that dual-trigger schemes can increase the number of oocytes collected and mature oocytes, the authors reached an agreement. They found that the dual-trigger group showed significantly higher CPR and LBR than the hCG trigger-alone group under the GnRH antagonist protocol [40–42], while Zhang et al. found no statistically significant differences for CPR or LBR using the progesterone-primed ovarian stimulation protocol [39]. The different views may be associated with the choice of the ovarian stimulation protocol. For the HORs, a retrospective study that included the patients with peak E_2_ < 4000 pg/mL demonstrated the superiority of dual-trigger cycles over GnRH agonist trigger alone not only in CPR but also in LBR [43]. Li and coworkers concluded that dual-trigger was associated with a lower incidence of severe OHSS than hCG trigger cycles while still maintaining a high-quality embryo rate [44]. The conclusion that the trigger mode does not affect the incidence of OHSS is not generalizable because the ovarian response of the included patients was not restricted and classified in this study.

The strength of our study is that we used PSM analysis to control for potential confounders between the two groups, thus making the outcomes independent from the different baseline characteristics. Moreover, because of the single-center design of this study, all IVF/ICSI cycles were carried out under uniform conditions, and embryos were cultured in the same media using the same techniques by the same embryologists. This approach minimized observational biases and the influence of varying culture media. However, this study has some limitations. The retrospective design is inevitably subject to selection bias, which together with the small sample size constitutes the main limitation of this study, leading to the conclusion that age was not a factor influencing EMR and MR. Also, propensity scores are not a substitute for randomization and would reduce the sample size, increasing the risk of type II error. There may still be potential variables that were not taken into account in the regression analysis, which could also contribute to biased results. Despite the insufficient evidence in this study, the trend we observed of higher MR in the dual-trigger group would provide a topic of interest for future studies. Hence, future studies should employ an RCT design in a larger sample size to verify and validate our results.

## Conclusion

The results of the dual-trigger method using GnRH agonist and hCG versus the hCG trigger alone for final oocyte maturation were similar with respect to the number of oocytes retrieved, number of embryos available, number of high-quality embryos, and normal fertilization rate for patients who were undergoing GnRH antagonist IVF/ICSI with fresh ET cycles. The MR was higher in the dual-trigger than hCG trigger-alone group, but binary logistic regression analysis showed that the trigger protocol was not a risk factor for miscarriage, a conclusion for which more evidence is needed. Other clinical benefits were not observed in terms of the OHSS incidence, IR, BPR, CPR, EMR, and LBR by excluding heterogeneous factors after PSM. In future clinical trials, we need to conduct well-designed prospective studies and focus on the potency ratio of both protocols in terms of cost-effectiveness, following the principles of individualization and optimization.

## Data Availability

The datasets generated and/or analyzed in this study are not publicly available for legal or ethical reasons, but will be made available by the corresponding author (Fang Lian: lianfangbangong@163.com) upon a reasonable request.

## Abbreviations

AMH: Anti-Müllerian hormone
BMI: Body mass index
CI: Confidence interval
COH: Controlled ovarian hyperstimulation
CPR: Clinical pregnancy rate
E_2_: Estrogen
EPR: Ectopic pregnancy rate
ET: Embryo transfer
FSH: Follicle-stimulating hormone
Gn: Gonadotrophin
GnRH: Gonadotropin-releasing hormone
hCG: Human chorionic gonadotropin
HOR: High ovarian responder
ICSI: Intracytoplasmic sperm injection
IR: Implantation rate
IVF: In vitro fertilization
LBR: Live birth rate
LH: Luteinizing hormone
LPS: Luteal phase support
MR: Miscarriage rate
OHSS: Ovarian hyperstimulation syndrome
OR: Odds ratio
P: Progesterone
POR: Poor ovarian responder
PSM: Propensity score matching
RCTs: Randomized controlled trials
